# Brain Metastases from Colorectal Cancer: A Systematic Review of the Literature and Meta-Analysis to Establish a Guideline for Daily Treatment

**DOI:** 10.3390/cancers13040900

**Published:** 2021-02-21

**Authors:** Sophie Müller, Franziska Köhler, Anne Hendricks, Carolin Kastner, Kevin Börner, Johannes Diers, Johan F. Lock, Bernhard Petritsch, Christoph-Thomas Germer, Armin Wiegering

**Affiliations:** 1 Department of General, University Hospital Wuerzburg, Visceral, Transplant, Vascular and Paediatric Surgery, 97084 Wuerzburg, Germany; mueller_s27@ukw.de (S.M.); koehler_f2@ukw.de (F.K.); berg_a2@ukw.de (A.H.); kastner_c@ukw.de (C.K.); boerner_k1@ukw.de (K.B.); johannes.diers@gmx.de (J.D.); lock_j@ukw.de (J.F.L.); germer_c@ukw.de (C.-T.G.); 2 Department of Radiology, University Hospital Wuerzburg, 97084 Wuerzburg, Germany; petritsch_b@ukw.de; 3 Comprehensive Cancer Centre Mainfranken, University of Wuerzburg, 97084 Wuerzburg, Germany; 4 Theodor Boveri Institute, Biocenter, University of Wuerzburg, 97084 Wuerzburg, Germany

**Keywords:** brain metastases, cerebral metastases, BM, colorectal cancer, CRC, systematic review, meta-analysis

## Abstract

**Simple Summary:**

Brain metastases (BM) from colorectal cancer (CRC) are rare. There is little available information regarding incidence, risk factors, prognostic factors, treatment, and overall survival (OS). In this systematic review we performed a research of the current literature and exposed an average incidence of 2.10%. The most-reported risk factors for developing BM were *KRAS* mutations and lung metastases. The majority of patients with brain metastases did not show neurological symptoms. Treatment options included surgery, radiation, or chemotherapy. While patients who received surgery had prolonged survival, the best survival time was found with a multimodality treatment regimen including neurosurgery.

**Abstract:**

Colorectal cancer (CRC) is the third most common malignancy worldwide. Most patients with metastatic CRC develop liver or lung metastases, while a minority suffer from brain metastases. There is little information available regarding the presentation, treatment, and overall survival of brain metastases (BM) from CRC. This systematic review and meta-analysis includes data collected from three major databases (PubMed, Cochrane, and Embase) based on the key words “brain”, “metastas*”, “tumor”, “colorectal”, “cancer”, and “malignancy”. In total, 1318 articles were identified in the search and 86 studies matched the inclusion criteria. The incidence of BM varied between 0.1% and 11.5%. Most patients developed metastases at other sites prior to developing BM. Lung metastases and *KRAS* mutations were described as risk factors for additional BM. Patients with BM suffered from various symptoms, but up to 96.8% of BM patients were asymptomatic at the time of BM diagnosis. Median survival time ranged from 2 to 9.6 months, and overall survival (OS) increased up to 41.1 months in patients on a multimodal therapy regimen. Several factors including age, blood levels of carcinoembryonic antigen (CEA), multiple metastases sites, number of brain lesions, and presence of the *KRAS* mutation were predictors of OS. For BM diagnosis, MRI was considered to be state of the art. Treatment consisted of a combination of surgery, radiation, or systemic treatment.

## 1. Introduction

Colorectal cancer (CRC) is the third most common type of malignant tumor worldwide, and in 2018, 880,792 deaths were reported due to CRC worldwide [1]. The incidence of CRC increases in an age-dependent manner, with the average age being 72–76 years at diagnosis [2]. Men are more frequently affected than women (23.6 cases vs. 16.3 cases per 100,000) [3]. Approximately 25% of patients present with distant metastases at time of diagnosis and another 25% will suffer from metastases further on [4]. Hepatic and pulmonary metastases are most common, while fewer patients develop brain metastases (BM). To date, there are standardized therapeutic pathways for the treatment of hepatic and pulmonary metastases which recommend surgical resection if complete resection is achievable. If the complete resection of metastases is not a therapeutic option, neoadjuvant chemotherapy is recommended with re-evaluation for surgery later on [5]. As BM are rare, there is a lack of data regarding management, with no guidelines for patients suffering from BM. Moreover, there seems to be a critical lack of information on presentation, treatment, and overall survival (OS) with regard to BM from CRC.

The aim of this systematic literature review and meta-analysis is to evaluate the incidence, common symptoms, overall survival, risk factors, and treatment strategies for BM due to CRC. Furthermore, based on the current literature we propose a clinical guideline for screening and treatment of BM from CRC.

## 2. Methods

**Figure 1:**
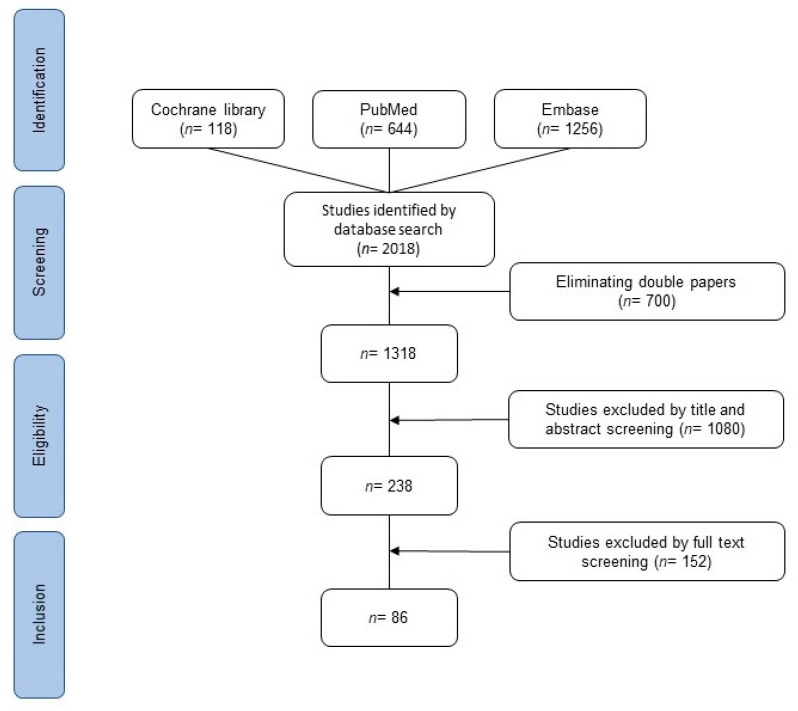
PRISMA flowchart of the search strategy.

We searched PubMed database, Embase database, and Cochrane database on 30 November 2020. All types of studies published between 1 January 2000 and 30 November 2020 were included. Studies with available abstracts in German or English were included. Search terms included “brain”, “metastas*”, “tumor”, “colorectal”, “cancer”, and “malignancy” using “and” or “or”. All patients with BM from CRC were included in the analyses. Duplicates were automatically removed by the literature organization program in addition to manual control. Two independent reviewers (SM, FK) performed the screening of titles and abstracts of all studies. Potentially relevant articles were reviewed in full to determine eligibility for inclusion. Any disagreement on manuscripts was discussed and solved by consensus. The selection process can be seen in the PRISMA (TRANSPARENT REPORTING of SYSTEMATIC REVIEWS and META-ANALYSES) flowchart (Figure 1) [6]. In the case that two studies examined the same study population, the more recent study was included. The literature organization was performed with Endnote20. Charts and tables were created with Microsoft Word, Microsoft PowerPoint, and RevMan5. Statistical analysis was performed with SPSS26 and RevMan5. As a measure of effects, the odds ratio (OR) with the corresponding 95% confidence interval (95%CI) was calculated. Statistical heterogeneity was assessed by calculating the chi^2^ and I^2^ statistics.

## 3. Results

The database search identified 2018 articles. After removing duplicates, 1318 articles were left for further investigation. After screening by title and abstract for suitability, 328 manuscripts were left. Articles were read in full text to check for inclusion criteria. Eighty-six papers matched the inclusion criteria and were used to perform the meta-analysis. None of these were randomized controlled trials (RCTs), as they mostly involved retrospective analyses. Articles were grouped in different categories to perform the meta-analysis (Table 1).

### 3.1. Incidence

**Figure 2:**
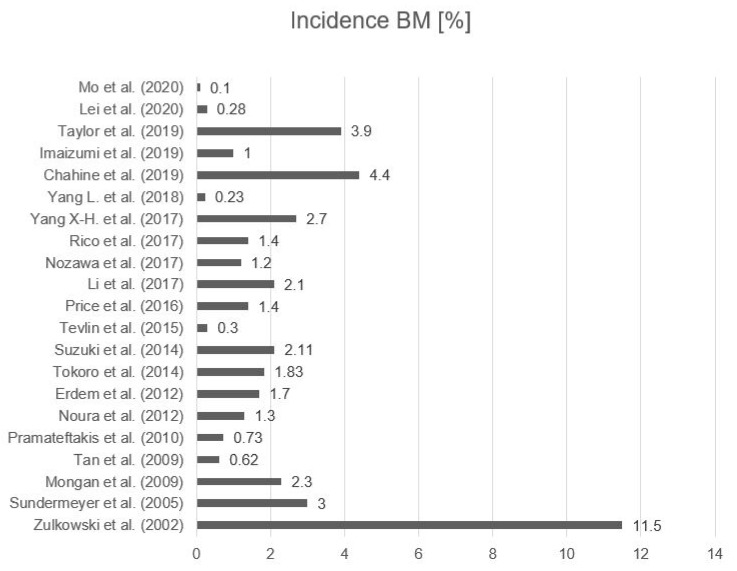
Incidence of BM (%) in all patients suffering from colorectal cancer (CRC) [7,8,9,10,11,12,13,14,15,16,17,18,19,20,21,22,23,24,25,26,27]. The overall average incidence of BM in CRC patients was 2.10% (95% confidence interval (CI) 0.98–3.22).

We identified 21 studies that reported on the incidence of BM due to CRC. Overall, 541,244 CRC cases were included, involving 1547 patients diagnosed with BM [7,8,9,10,11,12,13,14,15,16,17,18,19,20,21,22,23,24]. The overall average incidence of BM in CRC patients was 2.10% (95%CI 0.98–3.22) ranging from 0.1% up to 11.5% [7,8,9,10,11,12,13,14,15,16,17,18,19,20,21,22,23,24,25,26,27]. Fifty-seven percent of patients were male and 43% were female (Figure 2 and Table 2). Two studies focused on subgroup analysis and are not included in the overall evaluation of incidence. In patients with metastatic CRC, Shindorf et al. described a BM incidence of 14.6% [28]. McGovern et al. divided patients into ethic subgroups and discovered an incidence of 7% in their Asian subpopulation, while other ethnicities had an incidence ranging from 0.6% to 3.2% [29].

### 3.2. Symptoms

Six studies described symptoms in patients with BM from CRC. The initial symptoms of brain metastases were highly variable and were mostly not described in further detail. The most commonly reported symptoms were epileptic seizures, signs of increased intracranial pressure, or neurological symptoms [30,31,32,33,34,35]. Some patients did not show any symptoms at the time of diagnosis (Table 3). While Berghoff et al. found a ratio of 96.8% of asymptomatic patients, Kim D. et al. described only 5.3% of patients as being without symptoms [31,34]. Shindorf et al. performed a study that screened patients with metastatic CRC for BM, regardless of whether neurological symptoms were present. They showed that 76% of the patients with BM were asymptomatic [28].

### 3.3. Diagnostic Techniques

**Figure 3:**
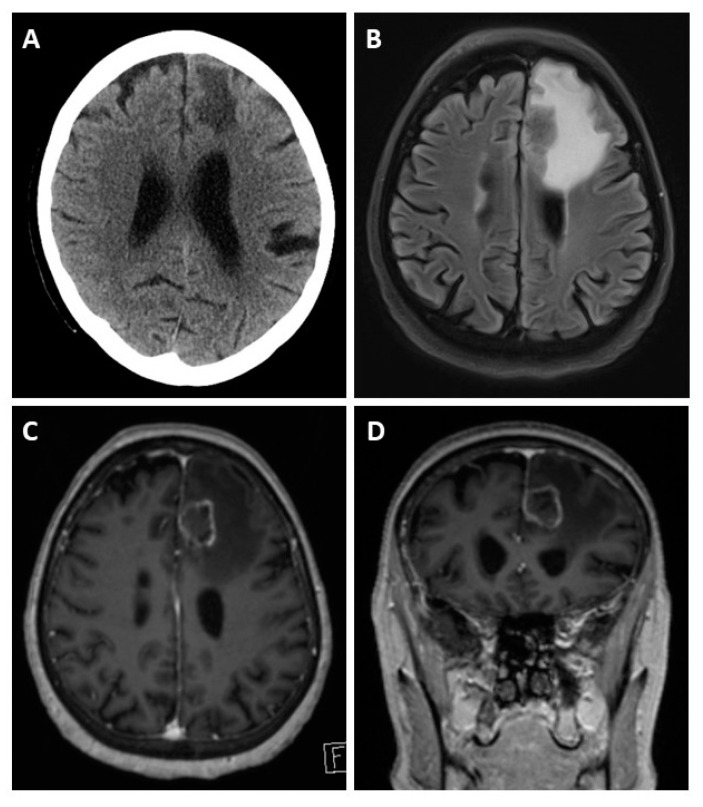
Cerebral CT and MRI scans of a patient with BM, a 62-year-old patient presenting with left frontal edema in computed tomography (**A**). The MRI scan reveals the actual extent of the edema in the fluid-attenuated inversion recovery (FLAIR) sequence (**B**). T1-weighted gadolinium post-contrast images in the axial (**C**) and coronal (**D**) orientation reveal causative left-frontal CRC metastasis.

Possible imaging modalities for diagnosis of BM are CT, MRI, or PET-CT. FDG-PET-CT is commonly performed as a whole-body examination, in which BM can appear as an incidental finding. Screening for BM is usually performed with cranial MRI [36]. We identified two studies that compared whole-body PET-CT to whole-body MRI to detect metastases from CRC. In these studies, PET-CT was superior for identifying lymph node metastases, for example locoregional to the primary tumor, whereas MRI was superior for detecting lesions of <1cm, especially BM [37,38]. As even smaller lesions and a meningeal carcinomatosis can be missed by imaging, diagnostic spinal fluid examination is proposed to identify tumor cells or DNA [39,40] (see Figure 3).

### 3.4. Prognostic Factors

#### 3.4.1. Overall

Nineteen studies depicted risk factors for developing BM; these studies are listed in Table 4. The majority of studies described an association of BM with lung metastases (LM) [13,21,22,41,42,43,44,45,46,47] or *KRAS* mutations [45,48,49,50,51]. Three studies reported that multiple extra cerebral metastases were related to the developing BM. Besides LM, bone metastases in particular were described as a risk factor [8,47,52,53]. Mo et al. and Yang X.-H. et al. identified a positive carcinoembryonic antigen (CEA) level as a risk factor for developing BM [7,13]. The primary CRC side might also have an association with an increased risk of developing BM. Prasanna et al., Yang X.-H. et al., and Christensen et al. reported a higher association between rectal cancer and BM [13,41,53]. Liu et al. described a correlation between *KRAS* mutations as well as BRAF mutations and BM, while Lee et al. depicted an association between ALK-translocation and BM [48,49]. In 2009 Mongan et al. described an association between BM development and chemokine receptor type 4 (CXCR4) [21].

#### 3.4.2. Lung Metastases

**Figure 4:**
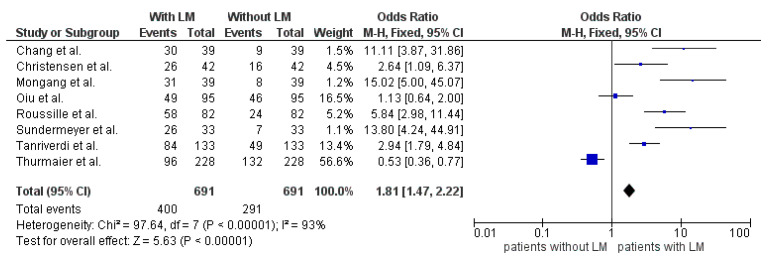
Forest plot comparison of BM patients with LM and without LM.

Eleven studies evaluated LM as a risk factor for developing brain metastases and reported a positive correlation (Table 5). In three of these studies only the abstracts were available but no full-text manuscripts. Eight studies were available in full text and are summarized in Figure 4. Altogether, 691 patients were examined. Of these patients, 400 had LM at diagnosis of BM, leaving 291 without LM at diagnosis of BM. The odds ratio was 1.81 (95%CI 1.47–2.22). Furthermore, a statistically significant difference was seen between the two groups (*p* <0.00001). The high heterogeneity may be caused by the small study populations (Figure 4).

#### 3.4.3. *KRAS* Mutation

**Figure 5:**
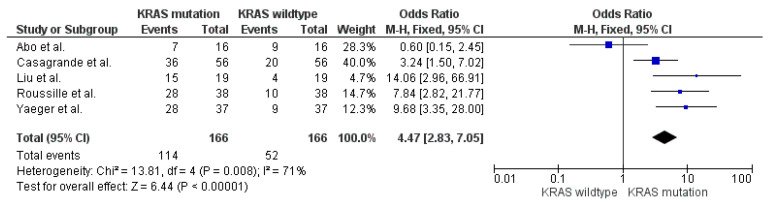
Forest plot comparison of BM patients with *KRAS* mutation and *KRAS* wild-type.

In five studies *KRAS* mutation was investigated as a risk factor for developing BM (Table 6). In one of them only the abstract was available. Five studies which reported on the *KRAS* mutation status of BM patients are shown in Figure 5. A total of 166 patients had a *KRAS* mutation analysis. In total, 114 patients with BM had a *KRAS* mutation (68%) and 52 patients had a *KRAS* wild-type (32%). The odds ratio was 4.47 (95%CI 2.83–7.05). The overall effect showed a significant difference (*p* <0.00001). The high heterogeneity may be caused by the small study populations (Figure 5).

### 3.5. Survival

**Figure 6:**
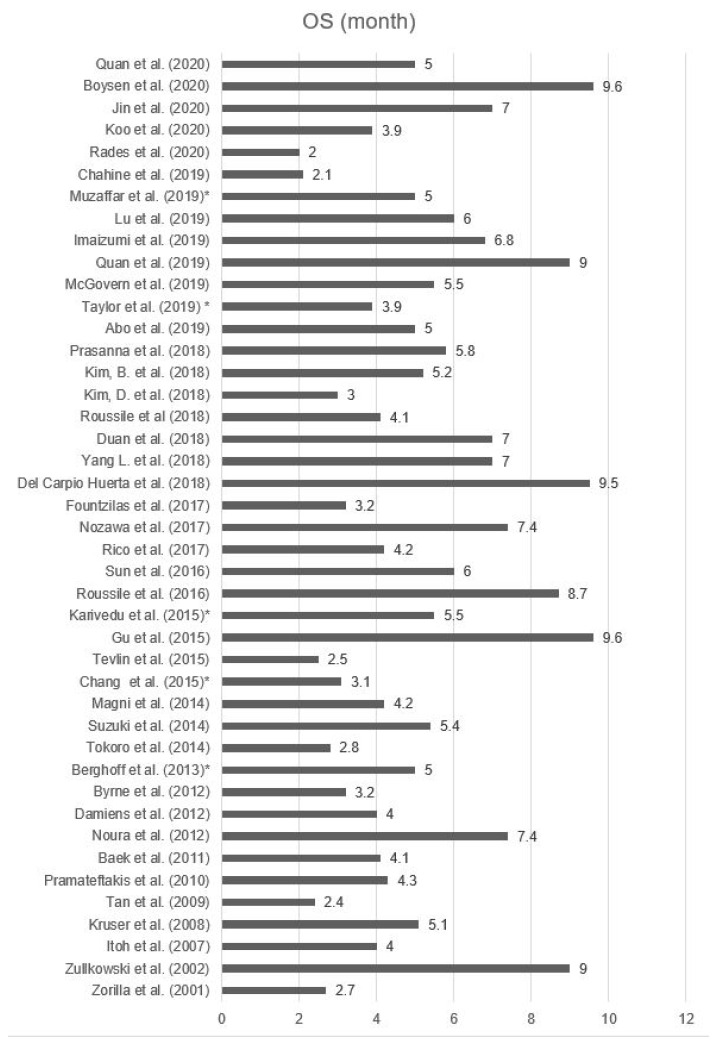
OS of patients with BM [9,10,11,12,14,18,20,23,24,25,26,27,29,34,45,46,53,54,55,56,57,58,59,60,61,62,63,64,65,66,67,68,69,70,71,72,73,74,75,76,77,78]. * only the abstract was available.

In 43 studies an overall survival (OS) with a range from 2 to 9.6 month from the time of BM diagnosis was determined. The median OS was 5.3 months (95%CI 4.6–5.9). In total there were 3611 patients with BM that were included in the OS analysis. The smallest study population reported on five patients, while the largest study included 475 patients with BM. The analyzed studies are shown in Figure 6 and Table 7 [9,10,11,12,14,15,18,20,23,24,25,26,27,29,34,45,46,53,54,55,56,57,58,59,60,61,62,63,64,65,66,67,68,69,70,71,72,73,74,75,76,77,78].

Twenty-five studies investigated factors of poor OS in patients with BM (Table 8). The most common factors were advanced age, low Karnofsky performance status (KPS), and extracranial metastases, as well as multiple BM. Four studies described a significant reduction in OS in patients with advanced age. Two studies did not report the exact OS. Duan et al. (>65 years: 4 months; <65 years: 10 months) and Quan et al. (>60 years: 4 months; <60 years: 8 months) described a survival benefit from 4 to 6 months [7,55,57,64]. Five studies evaluated KPS as a risk factor for poor OS. Two studies did not provide further specification on the OS. Lu et al. (KPS >70: 11 months; KPS <70: 4 months), Quan et al. (KPS >70: 7 months; KPS <70: 3 months), and Sun et al. (KPS >70: 2 months; KPS <70: 8 months) reported a survival benefit of 4–7 months [10,61,62,67,69]. Twelve studies investigated extracranial metastases as a prognostic factor for OS. Four did not report the exact OS. Three focused on a specific metastatic site and five evaluated extracranial metastases in general; these included the studies by Quan et al. (2020) (extracranial metastases: 4 months; no extracranial metastases: 6 months), Quan et al. (2019) (extracranial metastases: 7 months; no extracranial metastases: 28 months), Del Carpio Huerta et al. (extracranial metastases: 7.2 months; no extracranial metastases: 20.9 months), Gu et al. (extracranial metastases: 7 months; no extracranial metastases: 13 months), and Noura et al. (extracranial metastases: 8 months; no extracranial metastases: 24 months) [7,9,12,27,45,46,52,55,62,64,65,70]. Eleven studies reported multiple BM as a risk factor for poor OS. Four manuscripts did not provide the exact OS. Four studies compared one BM with more than one BM, those of Lu et al. (1: 9 months; >1: 5 months), Roussile et al. (2018) (1: 6.3 months, >1: 3.1 months), Roussile et al. (2016) (1: 12.3 months; >1: 4,9 months), and Gu et al. (1: 10 months; >1: 6 months). Duan et al. (1–2: 10 months; >2: 4 months), Sun et al. (1–2: 8 months; >2: 4 months), and Imaizumi et al. (1–3: 8.8 months; >3: 3.1 months) implemented different cut-offs [7,10,45,57,61,64,67,68,69,70,72]. Three studies reported that a positive CEA level was associated with a poor OS, but only Quan et al. reported a survival benefit of 3 months in patients with negative CEA levels [7,27,55]. 

Other reported risk factors for poor OS are the site of the primary tumor, N2 lymph node status, history of chemotherapy for the initial CRC, and association between the histological type. Chang et al. reported an association between poor OS and *KRAS* mutation (*KRAS* mutation: 22 months, *KRAS* wildtype: 36 months), Roussile et al. described *PDL1*+ as a predictor of poor OS (PDL1+: 1.8 months; PDL1–: 4.2 months) [46,68]. Quan et al., Mo et al., Kim B. et al., and Rades et al. developed a scoring system to predict the OS in patients with newly diagnosed BM [7,62,63,79]. These scores included common prognostic factors like age, KPS, CEA level, extracranial metastases, and number of BM, additionally grouping patients to predict the survival rates. Aprile et al. and Mitra et al. reported cases of HER2/neu positivity in BM from CRC, while the original tumor sample was HER2/neu-negative. HER2/neu expression might also be associated with a potential negative prognostic value in BM (HER2/neu+: 4.6 months, HER2/neu–: 6.5 months) [80,81].

### 3.6. Treatment

Altogether 18 studies evaluated different treatment modalities (Table 9). They investigated the influence of the treatment on the OS. Common therapies were radiation, surgery, chemotherapy, or a combination of the latter [9,14,25,26,27,34,54,57,61,62,64,67,68,70,73,74,84,85].

**Figure 7:**
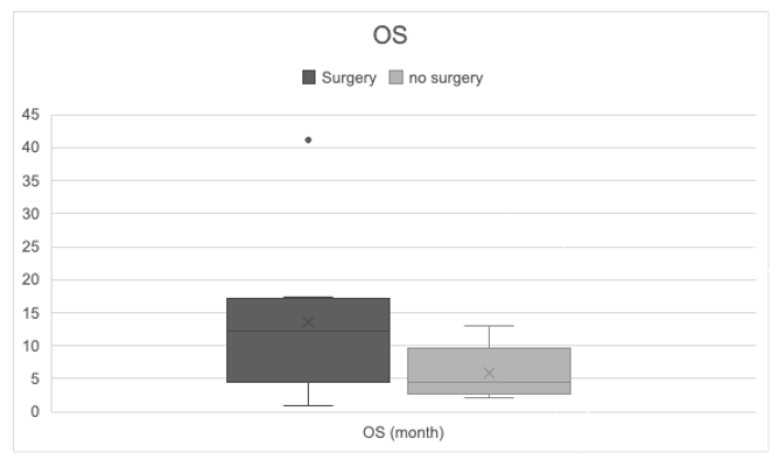
OS in patients with and without surgical intervention. Comparison of OS in patients with neurosurgery and without neurosurgery—median OS: surgery 11.69 months (95%CI 8.50–14.87); no surgery 5.28 months (95%CI 3.76–6.80). *t*-test *p* = 0.001.

**Figure 8:**
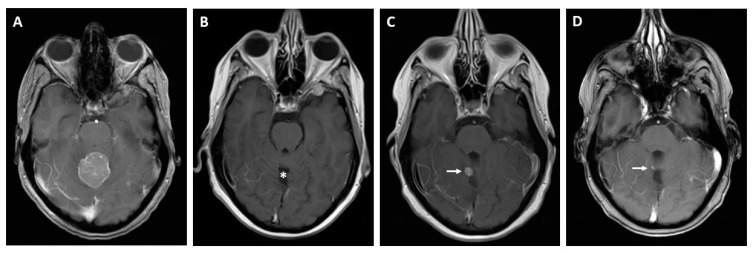
MRI scan before and after neurosurgery in a 46-year-old patient with a large histologically proven CRC metastasis in the vermis, prior to (**A**) and after neurosurgery (**B**) (asterisk: resection defect). Recurrence of a small local tumorous lesion (**C**) (arrow) 12 months later. Significantly declining tumor nodule after chemotherapy (**D**) (arrow).

Patients who underwent surgical resection with or without additional radiation or chemotherapy had a longer OS (11.69 months; 95%CI 8.50–14.87) compared to patients without surgery (5.28 months; 95%CI 3.76–6.80). The best survival rates were reported in patients who were treated with neurosurgery with/without radiation or chemotherapy. Jin et al. showed that a multimodal therapy regime resulted in a longer OS (41.1 months) (Figure 7 and Figure 8) [57].

In patients treated with best supportive care the OS was the lowest, at 0.43–2 months [26,57,64].

If surgery was not possible, a procedure for local control such as stereotactic radiosurgery or gamma-knife radiosurgery provided better OS in patients with 1–3 metastases [86,87,88,89,90]. With these procedures, local control of BM was possible in up to 95% of patients [90].

Finkelmeier et al. and Berghoff et al. reported that a combination of chemotherapy or radiation with bevacizumab prolonged survival rates and reduced neurological symptoms [91,92]. A recently published study by Amin et al. showed that immunotherapy in combination with radiation led to a longer survival of 34%, but no further information about the type of immunotherapy was provided [93].

## 4. Discussion

In this study we reviewed the current literature to analyze incidence, risk factors, treatment strategies, and overall survival in patients with BM from CRC.

Our systematic review confirmed that BM are rare in colorectal cancer patients. The incidence in the included studies ranged from 0.1% up to 11.5%. Zullkowski et al. described an incidence of 11.5% in their study population, which differed greatly from the other studies. This divergence might be due to their patient selection and the small study collective. In patients with metastatic disease, one study reported a BM incidence of 14.6% [28]. This matches our results, which showed an association of BM with extracranial metastases. The wide range of values for reported incidence might be due to the large number of asymptomatic BM patients. A lack of symptoms like nausea, vomiting, headaches, or reduced vision can lead to a late diagnosis.

Therefore, in studies with restricted cranial imaging brain metastases may be undetected, whereas in studies that perform cranial imaging more generously BM might be detected earlier.

Accordingly, studies that evaluated a screening program for BM described 96% of BM patients as being asymptomatic [28,31].

**Figure 9:**
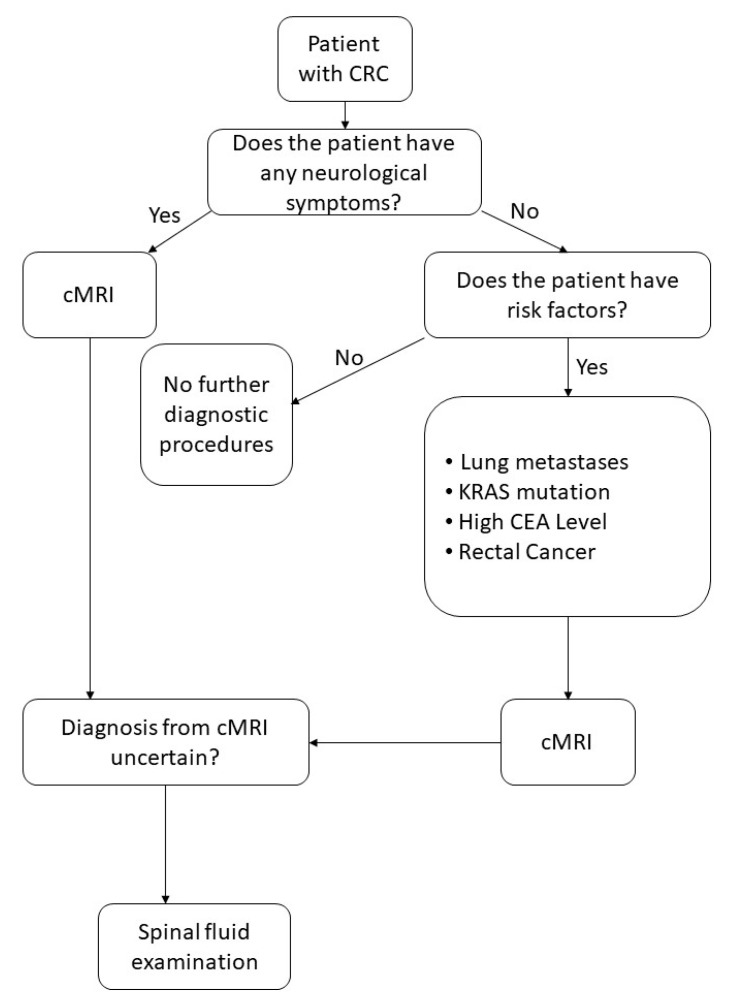
Assessment of screening for BM. (cMRI: cranial Magnetic resonance imaging)

Considering these findings, we propose a systematic screening program for CRC patients (Figure 9). Performing cranial imaging on every patient with CRC would not only lead to great number of physiological MRIs but would also be a financial burden for the health system. Therefore, we recommend cranial imaging in patients with symptoms or if risk factors are present (*KRAS* mutation, pulmonary metastases, rectal cancer, or positive CEA level). Our screening strategy is shown in Figure 9.

For other malignant diseases that more frequently lead to BM like breast cancer, studies proved higher survival rates and better treatment options by detecting BM early. Cagney et al. recommend screening for BM in patients with metastatic breast cancer [94] and Komorowski et al. reported that asymptomatic patients with metastatic breast cancer and HER2-overexpression profited from BM screening [95]. Morikawa et al. proved in their analysis that early detection of asymptomatic BM from breast cancer was associated with higher survival rates [96].

In patients with non-small-cell lung cancer the ESMO (EUROPEAN SOCIETY.

FOR MEDICAL ONCOLOGY) guidelines recommend brain imaging to screen for BM [97]. In comparison, the ESMO guidelines for metastatic CRC do not provide a recommendation regarding screening for BM. The ESMO guidelines for rectal cancer recommend cranial imaging in symptomatic patients [5,98]. Knowing that early diagnosis of BM in CRC leads to better survival rates, a screening program in patients with more than one risk factor for developing BM should be evaluated according to our recommended screening strategy (Figure 9).

A meta-analysis by Li et al. evaluated the diagnostic criteria for BM in lung cancer patients. Gadolinium-enhanced MRI had a higher sensitivity than 18FDG PET/PET-CT for the diagnosis of BM [99]. Pope et al. described the high sensitivity of cranial MRI in detection of BM independently of the primary tumors, and therefore recommended it as first choice for diagnosis as well as monitoring of therapy response [100]. In line with the results of this meta-analysis we would advise screening for BM from CRC with cranial MRI.

In case of BM, a number of risk factors affect the OS. In this analysis we found that a positive CEA level, a low KPS, and the presence of extracranial metastases and multiple BM predicted a poor OS. Edwards et al. evaluated the OS of elderly patients with various solid tumors. There was a great association between poor KFS and shorter OS [101]. Hwang et al. described, besides other factors, the influence of low KPS on the OS in metastatic cancer patients [102]. Furthermore, a few studies described an association between CEA level and survival after curative treatment for BM [103,104,105]. CEA may be suggestive of metastatic disease which is associated with poor OS [106,107,108].

**Figure 10:**
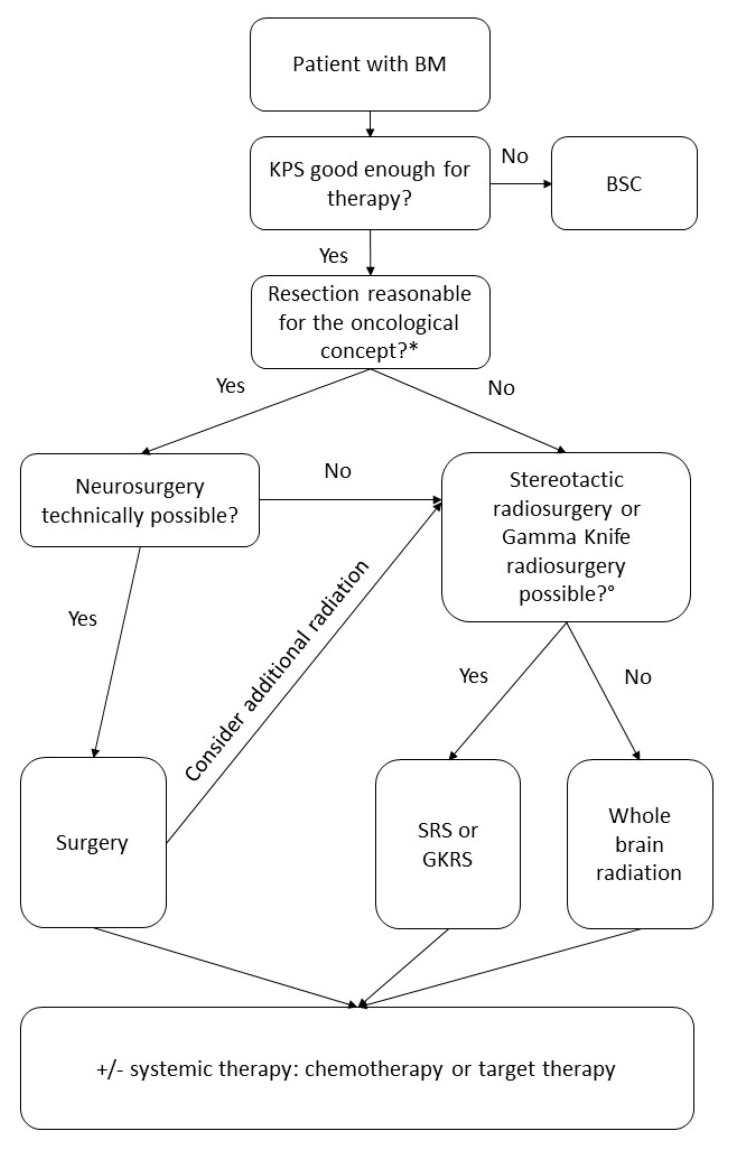
Assessment of therapy algorithm. BSC: best supportive care; SRS: stereotactic radiosurgery; GKRS: gamma knife radiosurgery; WBRT: whole-brain radiation. * Evaluate if neurosurgical resection is reasonable for the oncological therapeutic regime. The indication should be defined by an experienced neurosurgeon considering the size, number, and location of the metastases as well as symptomatology. ° The indication for SRS or GKRS should be considered individually for every patient. The DEGRO (Deutsche Gesellschafts für Radioonkologie) guidelines recommend SRS for a single BM <3 cm or 2–4 BM <2.5 cm for patients with life expectancy >3 months [109]. Lee et al. and Yamamoto et al. described how SRS for patients with up to 15 BM dependent on their position and size was associated with survival benefit and reduced risk of neurocognitive deterioration as compared to WBRT [110,111].

The best survival rates were found in patients with no extracranial metastases and a multimodal therapy regimen. If neurosurgical resection is possible, it leads to better OS rates if performed with additional radiation, chemotherapy, or targeted therapy. If neurosurgical resection is not possible, the number of BM is essential for defining the best treatment option. In patients with 1–3 BM, radiosurgery or gamma-knife radiosurgery is recommended, whereas patients with more than three BM should receive whole-brain radiation [86,87,88,89,90]. Our recommended treatment algorithm is shown in Figure 10.

This study has some limitations. Not all articles that were suitable by abstract screening were available in full text. We included them anyway in our analysis if adequate data were available in the abstract. Furthermore, all suitable studies were performed retrospectively, which could lead to a publication bias. The majority of studies included a low number of patients, as seen in the study overview in the appendix.

As BM are rare in patients with CRC, a number of studies lasted more than 10 years to reach the calculated study population. As immunotherapy has developed and changed rather quickly over the last decade, treatment modalities and recommendations might have changed during the study duration, which can also pose a risk of bias. The time interval of each included study is shown in the study overview in the appendix.

## 5. Conclusions

BM due to CRC represent a rare condition, but if patients develop BM, their overall survival is poor. The vast majority of patients (up to 96%) are asymptomatic, which can lead to late diagnosis. Therefore, we encourage the use of a screening program for patients with risk factors for developing BM. This way, BM can be detected early on and therapy options are superior. A multimodal treatment strategy provides the best OS, and can include surgery with/without radiation, chemotherapy, or targeted therapy. Nevertheless, new studies with a higher number of patients are necessary to obtain valid information about incidence, OS, and the best treatment strategies.

## Figures and Tables

**Table 1:** Categories and numbers of articles that matched the regarding category (numerous articles matched more than one category). OS: overall survival; BM: bone metastases.

Category	Articles Found
Incidence	21
Symptoms	7
Diagnosis	4
Risk factors for developing BM	17
Overall survival	43
Factors for poor OS	25
Treatment modalities	18

**Table 2:** Incidence and age distribution of patients with BM in included studies that reported on the number of patients with CRC, the number of patients with BM, the incidence of BM due to CRC, and percentage of male/female BM patients with BM [7,8,9,10,11,12,13,14,15,16,17,18,19,20,21,22,23,24,25,26,27]. * only the abstract was available.

Study	Number of CRC Patients	Number of BM Patients	Incidence (%)	Men	Women
Mo et al. (2020)	142,343	122	0.1	-	-
Lei et al. (2020)	192,923	532	0.28	-	-
Taylor et al. (2019)	1346	52	3.9	52%	48%
Imaizumi et al. (2019)	7147	68	1	63%	25%
Chahine et al. (2019) *	538	24	4.4	-	-
Yang L. et al. (2018)	170,793	401	0.23	51%	49%
Yang X.-H. et al. (2017) *	1104	30	2.7	-	-
Rico et al. (2017)	4100	59	1.4	49%	51%
Nozawa et al. (2017)	2238	23	1.2	-	-
Li et al. (2017) *	1714	36	2.1	58%	42%
Price et al. (2016)	4100	59	1.4	49%	51%
Tevlin et al. (2015)	4219	11	0.3	-	-
Suzuki et al. (2014)	5345	113	2.11	-	-
Tokoro et al. (2014)	1364	25	1.83	52%	48%
Erdem et al. (2012)	878	15	1.7	-	-
Noura et al. (2012)	2299	29	1.3	79%	21%
Pramateftakis et al. (2010)	670	5	0.73	-	-
Tan et al. (2009)	4378	27	0.62	52%	48%
Mongan et al. (2009)	1620	39	2.3	54%	46%
Sundermeyer et al. (2005)	1020	33	3.2	-	-
Zullkowskie et al. (2002)	113	13	11.5	-	-
Total	550,252	1716	2.10	57%	43%

**Table 3:** Clinical characteristics of BM in studies analyzing asymptomatic patients with BM from CRC [28,30,31,32,33,34,35] (° no information about asymptomatic patients).

Study	Number of BM Patients	Number and % of Asymptomatic Patients	Number and % with Epileptic Seizures
Berghoff et al. (2016)	224	210 (96.8%)	36 (14.6%)
Kim D. et al. (2018)	19	1 (5.3%)	1 (5.3%)
Shindorf et al. (2020)	25	19 (76%)	-
Goto et al. (2014)	-	-	-
Nemec et al. (2017)	-	-	-
Hassan et al. (2018)	-	-	-
Total	268	230 (85.8%)	-

**Table 4:** Risk factors for developing BM. UICC: Union internationale contre le cancer; TNM: TNM classification; CEA: carcinoembryogenic antigen; CXCR4: chemokine receptor type 4 [7,8,13,21,22,29,41,42,43,44,45,46,47,48,49,50,51,52,53]. * only the abstract was available.

	CEA Level	Staging (TNM or UICC)	Multiple Extra-Cerebral Metastases	Location of CRC	Bone Metastases	Lung Metastases	*KRAS*	Others
Mo et al. (2020)	x	High N or High T						
Lei et al. (2020)		UICC > III	x					
Thurmaier et al. (2020)		UICC IV	x		x	x		
McGovern et al. (2019) *								Asian ethnicity
Prasanna et al. (2018)				Rectal cancer	x			
Roussille et al. (2018)						x	x	
Liu et al. (2018)							x	*BRAF*
Lee et al. (2017) *							x	*ALK*
Yang X.-H. et al. (2017) *	x			Rectal cancer		x		
Christensen et al. (2016)				Rectal cancer		x		
Qiu et al. (2015)						x		
Casagrande et al. (2015)							x	
Yaeger et al. (2015)							x	
Chang et al. (2015) *						x		
Tanriverdi et al. (2014) *						x		
Zoratto et al. (2013) *						x	x	
Dhingani et al. (2012) *		UICC IV	x			x		
Mongan et al. (2009)				Left-sided CRC		x		*CXCR4*
Sundermeyer et al. (2005)						x		

**Table 5:** Number of BM patients with and without lung metastases (LM) in association with BM patients. * only the abstract was available [13,21,22,41,42,43,44,45,46,47,52].

	Number of CRC Patients	Number of BM Patients	Number and % of BM and LM	Number and % of BM, no LM	Number of LM Patients
Thurmaier et al. (2020)	-	228	96 (42.1%)	132 (57.9%)	-
Roussille et al. (2018)	-	82	58 (72%)	24 (28%)	-
Yang X.-H. et al. (2017) *	1104	30	-	-	-
Christensen et al. (2016)	480	42	26 (62%)	16 (38%)	156
Qiu et al. (2015)	46,027	95	49 (51.6%)	46 (48.4%)	1750
Chang et al. (2015) *	-	39	30 (76.9%)	9 (23.1%)	-
Tanriverdi et al. (2014) *	4864	133	84 (74%)	49 (26%)	-
Zoratto et al. (2013) *	623	26	-	-	-
Dhingani et al. (2012) *	301	52	-	-	-
Mongang et al. (2009)	1620	39	31 (78%)	8 (22%)	-
Sundermeyer et al. (2005)	1020	33	26 (78.8%)	7 (21.2%)	422
Total	56,039	799	-	-	2328

**Table 6:** Number of BM patients with *KRAS* mutation and *KRAS* wild-type in association with BM patients. * only the abstract was available [45,48,49,50,51,54].

	Number of CRC Patients	Number of BM Patients	Number and % of BM + *KRAS* Mutations	Number and % of BM + *KRAS* Wild-Type
Abo et al. (2019) *	-	16	7 (43.7%)	9 (56.3%)
Roussile et al. (2018)	-	38	28 (74%)	10 (26%)
Liu et al. (2018)	461	19	15 (78.9%)	4 (21.1%)
Lee et al. (2017) *	-	11	-	-
Yaeger et al. (2015)	-	37	28 (75.7%)	9 (24.3%)
Casagrande et al. (2015)	-	56	36 (64.3%)	20 (35.7%)

**Table 7:** Study characteristics and OS. Studies reported OS in months, the number of patients with CRC, and the number of patients with BM from CRC [9,10,11,12,14,18,20,23,24,25,26,27,29,34,45,46,53,54,55,56,57,58,59,60,61,62,63,64,65,66,67,68,69,70,71,72,73,74,75,76,77,78]. *only the abstract was available.

	OS (Months)	Number of CRC Patients	Number of BM Patients
Quan et al. (2020)	5	-	371
Boysen et al. (2020)	9.6	38,131	235
Jin et al. (2020) *	7	>30,000	104
Koo et al. (2020)	3.9	-	106
Rades et al. (2020)	2	-	57
Chahine et al. (2019) *	2.1	538	24
Muzaffar et al. (2019) *	5	-	475
Lu et al. (2019)	6	-	80
Imaizumi et al. (2019)	6.8	7147	68
Quan et al. (2019)	9	-	52
McGovern et al. (2019) *	5.5	76	5
Taylor et al. (2019) *	3.9	-	52
Abo et al. (2019) *	5	-	16
Prasanna et al. (2018)	5.8	5967	109
Kim B. et al. (2018) *	5.2	-	107
Kim D. et al. (2018)	3	-	19
Roussile et al. (2018)	4.1	-	82
Duan et al. (2018)	7	-	78
Yang L. et al. (2018)	7	170,793	401
Del Carpio Huerta et al. (2018)	9.5	-	28
Fountzilas et al. (2017)	3.2	-	40
Nozawa et al. (2017)	7.4	2238	23
Rico et al. (2017)	4.2	4100	59
Sun et al. (2016) *	6	-	45
Roussile et al. (2016) *	8.7	-	135
Karivedu et al. (2015) *	5.5	-	94
Gu et al. (2015)	9.6	-	93
Tevlin et al. (2015)	2.5	4219	11
Chang et al. (2015) *	3.1	-	39
Magni et al. (2014)	4.2	-	41
Suzuki et al. (2014)	5.4	5345	113
Tokoro et al. (2014)	28	1364	25
Berghoff et al. (2013) *	5	-	69
Byrne et al. (2012)	3.2	1304	52
Damiens et al. (2012)	4	-	48
Noura et al. (2012)	7.4	2299	29
Baek et al. (2011)	4.1	-	118
Pramateftakis et al. (2010)	4.3	670	5
Tan et al. (2009)	2.4	4378	27
Kruser et al. (2008)	5.1	-	49
Itoh et al. (2007)	4	-	5
Zullkowski et al. (2002)	9	113	13
Zorilla et al. (2001)	2.7	-	9
Total	5.3	>278,682	3611

**Table 8:** Factors for poor OS [7,9,10,12,27,45,46,52,55,56,57,59,60,61,62,63,64,65,67,68,69,70,72,82,83]. * only the abstract was available. KPS: Karnofsky performance status.

	Positive CEA level	Low KPS	Extracranial Metastases	Multiple BM	Age	Location of CRC	Others	Score
Thurmaier et al. (2020)			Liver					
Quan et al. (2020)	x		x		x			x
Mo et al. (2020)	x		x	x	x			x
Boysen et al. (2020)							N2	
Jin et al. (2020) *				x	x			
Rades et al. (2020)								x
Muzaffar et al. (2019) *						x		
Lu et al. (2019)		x		x				
Imaizumi et al. (2019)		x		x			History of chemotherapy	
Quan et al. (2019)		x	x					
Taylor et al. (2019) *			Liver					
Kim B. et al. (2018) *								x
Roussile et al. (2018)			Lung	x			PDL1+	
Duan et al. (2018)			Bone	x	x			
Yang L. et al. (2018)			x			x	Pathology	
Del Carpio Huerta et al. (2018)			x			x		
Berghoff et al. (2017) *						x		
Sun et al. (2016) *		x		x				
Nieder et al. (2016)								x
Roussile et al. (2016) *				x				
Karivedu et al. (2015) *		x		x				
Gu et al. (2015)			x	x				
Chang et al. (2015) *			x				*KRAS* mutation	
Berghoff et al. (2013) *				x		x		
Noura et al. (2012)	x		x					

**Table 9:** OS in months for different treatment modalities [9,14,25,26,27,34,54,57,61,62,64,67,68,70,73,74,84,85]. * only the abstract was available. BSC: best supportive care. Rx: radiation therapy. Cx: chemotherapy. OP: surgery

	OS (month)	BSC	Rx	Cx	Rx + Cx	Op	Op + Rx	OP + Cx	OP + Rx + Cx
Jin et al. (2020) *	7	0.43	3.13	-	12.2	4.8	14	-	41.1
Lu et al. (2019)	6	-	3	5	10	10	-	17	-
Quan et al. (2019)	9	-	7	13	-	17	17	-	-
Taylor et al. (2019) *	3.9	-	-	-	-	1	4.4	-	12.3
Abo et al. (2019) *	5	-	-	-	-	-	17.4	-	-
Kim D. et al. (2018)	3	-	2.5	-	-	-	5	-	-
Duan et al. (2018)	7	2	-	-	-	-	-	-	14.1
Del Carpio Huerta et al. (2018)	9.5	-	4.6	-	-	-	12.1	-	-
Rico et al. (2017)	4.2	-	2.2	-	-	-	8.5	-	-
Sun et al. (2016) *	6	-	4	4	10	12	-	-	-
Roussile et al. (2016) *	8.7	-	4.9	-	-	14.8	-	-	-
Gu et al. (2015)	9.6	-	-	-	-	11	15.5	-	-
Suzuki et al. (2014)	5.4	1.2	5.1	-	-	-	10.5	-	-
Tokoro et al. (2014)	2.8	1.5	1.5	-	-	4.8	-	-	-
Kim H. et al. (2013)	-	-	5.6	-	-	16.2	-	-	-
Byrne et al. (2012)	3.2	-	3.4	1.7	-	13.2	-	-	-
Damiens et al. (2012)	4	2	4	-	-	3	13	-	-
Noura et al. (2012)	7.4	-	7.9	-	-	5.1	11.4	-	-

## Data Availability

The data presented in this study are openly available in PubMed, Embase, or Cochrane library.
